# Introducing and applying Newtonian blurring: an augmented dataset of 126,000 human connectomes at braingraph.org

**DOI:** 10.1038/s41598-022-06697-4

**Published:** 2022-02-23

**Authors:** László Keresztes, Evelin Szögi, Bálint Varga, Vince Grolmusz

**Affiliations:** 1 PIT Bioinformatics Group, Eötvös University, 1117 Budapest, Hungary; 2 Uratim Ltd., 1118 Budapest, Hungary

**Keywords:** Computational biology and bioinformatics, Neuroscience, Neurology, Mathematics and computing

## Abstract

Gaussian blurring is a well-established method for image data augmentation: it may generate a large set of images from a small set of pictures for training and testing purposes for Artificial Intelligence (AI) applications. When we apply AI for non-imagelike biological data, hardly any related method exists. Here we introduce the “Newtonian blurring” in human braingraph (or connectome) augmentation: Started from a dataset of 1053 subjects from the public release of the Human Connectome Project, we first repeat a probabilistic weighted braingraph construction algorithm 10 times for describing the connections of distinct cerebral areas, then for every possible set of 7 of these graphs, delete the lower and upper extremes, and average the remaining 7 − 2 = 5 edge-weights for the data of each subject. This way we augment the 1053 graph-set to 120 $\times$ 1053 = 126,360 graphs. In augmentation techniques, it is an important requirement that no artificial additions should be introduced into the dataset. Gaussian blurring and also this Newtonian blurring satisfy this goal. The resulting dataset of 126,360 graphs, each in 5 resolutions (i.e., 631,800 graphs in total), is freely available at the site https://braingraph.org/cms/download-pit-group-connectomes/. Augmenting with Newtonian blurring may also be applicable in other non-image-related fields, where probabilistic processing and data averaging are implemented.

## Introduction

Gaussian blur operations^1–3^ are standard tools in image processing for different purposes. In Artificial Intelligence (AI), it is widely used for image-data augmentation, i.e., increasing the size of the training and testing image databases by blurring some parts of the original images and adding the resulting pictures to the dataset^4–6^. The augmented dataset is applicable for pattern recognition tasks with AI tools, and—since no new objects were artificially introduced, just some parts of the images blurred—the integrity and the validity of the image-set will not be hurt. Additionally, in many applications in pattern recognition, the positive or negative identification of patterns is desirable even when some noise (i.e., blurring) is present in the images.

Augmenting can also be applied successfully for the prevention of overfitting in the training of AI applications: introducing small perturbations in the data would help the AI tool to learn the “important” features of the input^7,8^.

In non-image datasets, and especially biological datasets, similar augmenting techniques are generally missing. In the present contribution, we describe an augmenting method, which is called “Newtonian blurring”, paying tribute to Isaac Newton’s Binomial Theorem and binomial coefficients^9^. The Newtonian blurring is introduced and first applied here for augmenting a set of human braingraphs.

### Human braingraphs

Human braingraphs (or connectomes) describe the cerebral connections between the anatomically identified areas of the human brain. The human brain, in a simplified setting, consists of gray matter and white matter. Gray matter is—again, with simplifications—formed from the bodies (or somas) of the neurons, and the white matter from the axonal fibers of the neurons: these fibers connect the somas of the neurons. The white matter also contains myelin covers of these fibers, providing electrical insulation of the axonal fibers. The gray matter is situated on the outer surface (the cortex) of the brain and also in some sub-cortical regions; the white matter can be found under the cortex, in the internal parts of the brain.

It is a very interesting question to map the neuronal level connections of the brain: in this mapping, a graph is defined, where the nodes correspond to neurons, and an edge connects two neurons if the axon of one of them is connected to the other’s dendrite. Unfortunately, no such graph can be measured for the human brain, which has 80 billion neurons: we simply do not have methods for discovering all the connections of such a great number of vertices. The nematode worm *Caenorhabditis elegans* is the only developed organism, for which such a graph is described^10^, but it has only 203 neurons. Very recently, it is announced that the neuronal level connectome of the fruit fly *Drosophila melanogaster*^11^ is within our reach in one or two years, but today only the connections between some 25,000 neurons are available from the total of 100,000 neurons of the fruit fly brain^12^.

The connectome (or the braingraph) of humans can be described today in a coarse resolution: the vertices correspond to around 1000 anatomically identified areas (called ROIs, Regions of Interest) of the gray matter. We write here “areas” instead of “volumes”, since, in the cortex, the gray matter is relatively thin, typically 2–4 mm thick. The edges of the graph connect those pairs of vertices, between which a magnetic resonance imaging (MRI) workflow discovers streamlines^13,14^. More exactly, diffusion MRI is capable of identifying the distribution of directional freedom of movement of water molecules in the brain, notably in and around neurons. This distribution is isotropic in the large body (soma) of the neurons, but it is directionally constrained (anisotropic) in and around the long, thin axons that are aligned with each other. Therefore, streamlines can be discovered in the white matter of the brain, and one can construct braingraphs (or connectomes) as follows: two vertices, corresponding to two ROIs, are connected by an edge if at least one streamline is discovered between them. The number of fibers detected is assigned as weights (fiber number) to the edges. For more details, we refer to Refs.^13,15–17^.

As a result of a large, NIH-funded research project, the Human Connectome Project (HCP)^18,19^, high-quality diffusion MRI data were published from hundreds of healthy, young adults. Applying this resource, together with an integrated toolset^20^ for computing braingraphs from the MRI data, our research group successfully introduced numerous mathematical and graph-theoretical techniques into the analysis of the human braingraphs^15,16,21–32^.

We have also published the braingraphs, computed by us at the site https://braingraph.org. First, we had made available several graph-sets, based on the HCP 500 Subjects Release^15,16,23^, later we have published 1064 human connectomes, each in five resolutions^17^, which were based on the 1200 Subjects Release of the Human Connectome Project. For each set and each resolution, the edges of the graphs are weighted by the fiber numbers detected between their endpoints.

The 1064 braingraphs, described in Ref.^17^, were computed by using probabilistic algorithms, and typically, any two runs on the same input yielded slightly different outputs. For increasing the robustness, and the reproducibility, we have applied an averaging and extreme-value deleting strategy, as follows.

### Basic averaging strategy

- For all subjects, the tractography step of the processing, which determined the streamlines, connecting the ROIs of the brain, was computed 10 times.
- For each subject and each resolution (i.e., 83, 129, 234, 463, and 1015 nodes), the braingraph of the subject was computed, and ten interim weights were assigned for each edge. The ten interim weights corresponded to the number of fibers detected in the 10 tractography runs, respectively.
- Those edges, which appeared with 0 fibers in at least one of the 10 tractography runs, were deleted.
- For the remaining edges, the maximum and minimum edge weights were deleted, and the remaining eight weights were averaged (by simple arithmetic mean). This value was assigned to the edge as its (final, non-interim) weight.

In Ref.^17^ the particular choice of 10 repetitions is analyzed and explained in detail.

In the present contribution, we modify the averaging strategy above and define the Newtonian blurring for braingraphs.

We remark that the averaging process called basic averaging strategy^17^ clearly increased the robustness of the results and decreased the variance of the graph weights due to the probabilistic processing. In the introduction of the Newtonian blurring, we also focus on robustness, and we do not intend to add any artifacts to the data: we just slightly perturb the error-correcting, averaging steps of the real, unmodified data.

We also note that even in the augmented graphs, we have used averaging error correction for increasing robustness.

## Discussion and results

### Newtonian blurring

Here, we describe the new Newtonian blurring method as a modification of the basic averaging strategy for braingraphs.

- For all subjects, the tractography step of the processing, which determined the streamlines, connecting the ROIs of the brain, is computed 10 times.
- For each subject and each resolution, the braingraph of the subject is computed, and ten interim weights were assigned for each edge. In other words, ten weighted graphs are generated for each subject and each resolution.
- For each subject and each resolution, 7 graphs from the 10 weighted graphs are chosen in every possible way (i.e., ${10\atopwithdelims ()7}=120$ ways).
- Those edges, which appeared with 0 fibers in at least one of the 7 chosen graphs, are deleted from all the 7 graphs.
- For each edge, the maximum and minimum edge weights out of the 7 are deleted, and the remaining five weights are averaged (by simple arithmetic mean). This value is assigned to the edge as its (final, non-interim) weight. In other words, from the 7 weighted graphs we prepared one graph in this workflow.

We emphasize the difference in the basic averaging strategy and the Newtonian blurring: in the former the ten tractography runs for a fixed subject and resolution result one graph, in the latter the ten tractography runs result 120 graphs.

We note that for a given subject and a given resolution, if a graph-edge “e” is present in all the 10 tractography runs with a non-0 fiber number, then “e” will be present in all the 120 augmented graphs as well.

If “e” is present with a 0 fiber number in exactly one tractography run out of the 10, then edge “e” will be deleted from the augmented graphs where the 0 weight is chosen, i.e., from $9\atopwithdelims ()6$ graphs, and will be present in the remaining $9\atopwithdelims ()7$ graphs (by the Pascal-triangle identity ${9\atopwithdelims ()6}+{9\atopwithdelims ()7}={10\atopwithdelims ()7}$).

This way, we augment the starting braingraph-set to a 120-times larger set. The augmented braingraphs do not contain any artificial components: they even contain strict error correction by deleting the extremal edge weights and averaging the remaining five weights.

We point out that Gaussian blurring in image processing introduces “artificial noise” in several parts of an image by—essentially—an intricate pixel-averaging strategy. We have two reasons for calling the new augmenting method “blurring”: The first is paying tribute to the Gaussian blurring method. The second is as follows: for each edge, instead of deleting the two extremes and averaging the remaining 8 weights, we choose all the possible 7 from the 10 weights, delete the extremes, and average the remaining 5 edge weights: that is, we use a data-cleaning method (i.e., averaging) even in the augmentation step, but not so strong as it were possible without the augmenting step. Without the augmenting step, 8 weights are averaged; with the augmenting step, only 5 weights are averaged. This weaker averaging strategy possibly corrects fewer inconsistencies in the measured data and, consequently, it could be seen as a “blurring” relative to the case when 8 weights are averaged.

### On the deviation of the augmented graphs

The following is a natural question: How diverse are the augmented graphs compared to the starting ones? Clearly, one needs certain diversity in the augmented set for the applications in artificial intelligence since simply repeating the starting graph several times is useless. However, as in the Gaussian blurring, where the blurred images are very similar to the original ones, here we can also find a similar situation: the Newtonian-blurred graphs are also similar to the starting one.

For a distance measure, we apply Jaccard-distance for the edges *E*(*G*) of graphs *G*:

$$\begin{aligned} J(G_1,G_2)={{|(E(G_1)\cup E(G_2))-(E(G_1)\cap E(G_2))|}\over |E(G_1)\cup E(G_2)|}, \end{aligned}$$

where |*A*| denotes the number of elements of set *A*. The Jaccard distance of any two graphs is a non-negative number between 0 and 1; it describes the fraction of edges in which the graphs differ.

**Figure 1:**
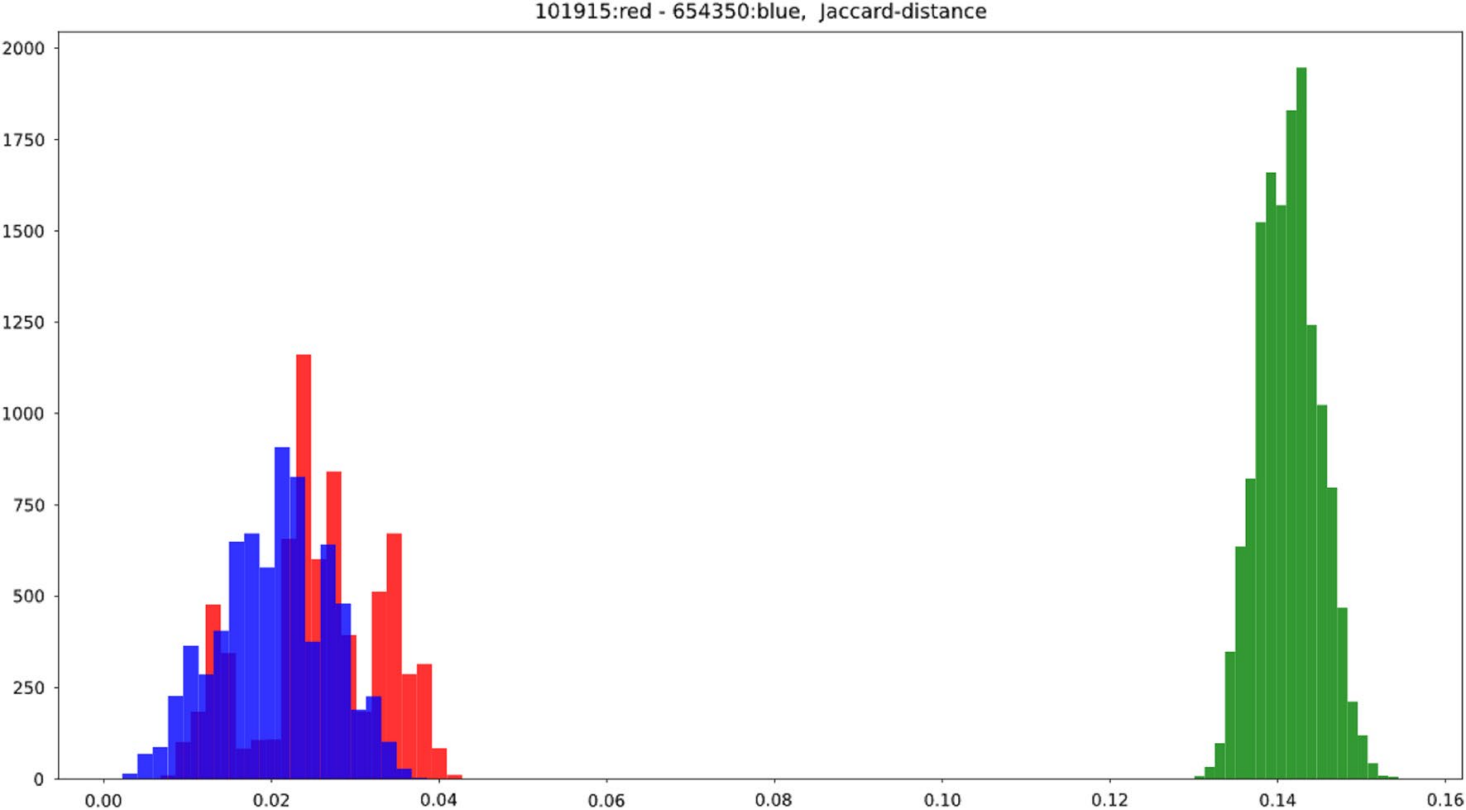
The distribution of the Jaccard-distances of all the pairs formed from the 120 augmented graphs of the two closest (measured in Jaccard distance) graphs from the 1053 ones: the graphs No. 101915 and 654350. From the 120 + 120 = 240 augmented graphs, we can form 28,680 pairs. The pairs are partitioned into three classes: Red class: both members of the pair belong to subject 101915; Blue class: both members of the pair belong to subject 654350; Green class: one member belongs to subject 101915, the other to 654350. On the x-axis, the Jaccard distance is given; on the y-axis, the count of the pairs of graphs with the given Jaccard distance is shown (it is a histogram).

Figure 1 depicts the distribution of the Jaccard-distances of all the pairs formed from the 120 augmented graphs of subjects 101915 and 654350, the two most-similar subjects as measured by Jaccard distance of their (basic averaging strategy) graphs.

We have chosen the most similar graphs in order to show that the augmenting technique not only changes the weights of the edges, but also the edge-sets even in the augmented versions of the two most similar graphs. The same finding from more different graphs would be less striking.

From the 120 + 120 = 240 graphs, we can form ${240\atopwithdelims ()2} = 28,680$ pairs. The pairs are partitioned into three classes in Fig. 1:

- *Red class* both members of the pair belong to subject 101915.
- *Blue class* both members of the pair belong to subject 654350.
- *Green class* one member belong to subject 101915, the other to 654350.

On the x-axis, the Jaccard distance is given; on the y-axis, the count of the pairs of graphs with the given Jaccard-distance (it is a histogram).

In the figure, one can observe that the red, blue, and green classes form Gaussian distributions. Additionally, the expectation of the fraction of the different edges within the 120 graphs of the same origins are a little larger than 0.02, while the expectation of the Jaccard distance in the green class is more than 0.14, i.e., at least 14% of the edges differ.

Therefore, typically, more than 2% of the edges differ in the graphs of the same subject and in more than 14% in graphs of different subjects.

### An application example with support vector machines

Here we concisely describe an application of our augmented dataset for training and testing linear Support Vector Machines (SVM)^33^, on a simple classification task of braingraphs.

A typical use of data augmentation is preventing the overfitting in the training of AI tools. Here we demonstrate an application of our augmented braingraph set in this aspect.

In the SVM classification, we have *k* elements from the *n*-dimensional real space, denoted by $x_1,x_2,\ldots ,x_k$, and *k* labels $y_1,y_2,\ldots ,y_k$, where for all *i* $y_i=1$ or $y_i=-\,1$, corresponding to a binary classification. The goal is finding the *n*-dimensional hyperspace, which “best” separates the $x_i$ points with $y_i=1$ from the $x_j$ points, where $y_j=-1$. The hyperspace is characterized by the equation $wx+b=0$, and ideally, the separation is performed by the sign of $wx+b$, that is, for all *i*, ${{\mathrm{sgn}}}(wx_i+b)=y_i$. From a short computation, the optimal hyperspace is characterized by *w* and *b*, satisfying

1 $$\begin{aligned} \min _{w,z,b}\left( {1\over 2}w\cdot w + C\sum _{i=1}^kz_i\right) , \end{aligned}$$

where $$z_i\ge 0$$ and $$y_i(wx_i-b)\ge 1-z_i$$.

Here, in (1), $C>0$, the amount of penalty for wrongly classifying a point. If in (1) we simply repeat all $x_i$’s 120 times, then we need to use *C*/120 instead of the original *C* if we want to retain the value of the minimum. In the augmented case, when all data points are exchanged to 120 new, augmented data points, we also need to use *C*/120 for comparisons with the original value of (1).

**Figure 2:**
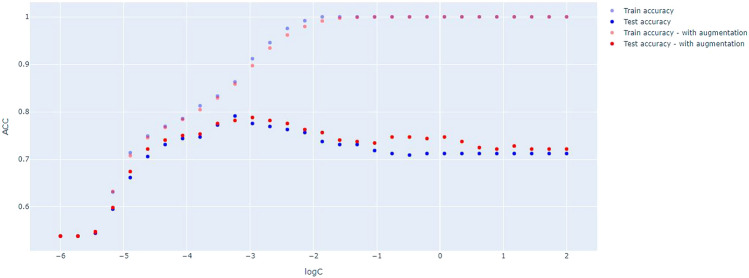
The comparison of train- and test-accuracies with and without augmentation, as the function of the logarithms of parameter C. The figure shows the better test accuracy in almost all domain of log C with augmentation.

Figure 2 shows the accuracy of applying our augmenting method for linear SVM training and testing for the prediction of the sex of the subjects in our original and augmented braingraph set as the function of $\log C$.

More exactly, in the non-augmented application, we have trained on 737 braingraphs (397 females and 340 males) and tested on 316 graphs (170 women and 146 men); we have applied the 83-node resolution, 0–1 weighted graphs in the example (we need to add that in our related work^34^ the fiber-number weighted graphs were used in the SVM classification).

In the augmented application, the original 737 training braingraphs were augmented 120-fold to 120 $\times$ 737 graphs, and the test set is identical to the non-augmented case, it contains 316 graphs.

We remark that a similar advantage of the augmented data set can be demonstrated by logistic regression classification.

## Conclusions

We have introduced a new augmenting method for non-image data, called Newtonian blurring. With the new method, we have prepared 631,800 augmented braingraphs for artificial intelligence applications (training and testing) and made the set publicly available at the https://braingraph.org/cms/download-pit-group-connectomes/. One important feature of the Newtonian blurring that it would not introduce any artifacts, any artificial perturbation into the data, and, still, it is capable of considerable augmenting for AI applications. Another important feature of the new method that it can be used for avoiding overfitting and improving classification accuracy, as we have shown in a Support Vector Machine application example.

## Data Availability

The data source of this work is published at the Human Connectome Project’s website at http://www.humanconnectome.org^18^. The parcellation data, containing the anatomically labeled ROIs, is listed in the CMTK nypipe GitHub repository https://github.com/LTS5/cmp_nipype/blob/master/cmtklib/data/parcellation/lausanne2008/ParcellationLausanne2008.xls. The braingraphs, computed by us, can be accessed at the http://braingraph.org/cms/download-pit-group-connectomes/ site. For each resolution, a compressed archive is available, containing 126,360 graphs. Technical remark: uncompressing an archive containing that number of files takes several minutes, even on fast systems.
